# Identification of Polysialic Acid and Chondroitin-like Polysaccharides of *Moraxella bovis* Strains Associated with Infectious Bovine Keratoconjunctivitis

**DOI:** 10.1021/acsinfecdis.5c00628

**Published:** 2025-12-04

**Authors:** Justine Vionnet, Dwight C. Peterson, John Dustin Loy, Emily Wynn, Marcos Daniel Battistel, Matthew Hille, Michael L. Clawson, Willie Vann

**Affiliations:** † Center for Biologics Evaluation and Research, Food and Drug Administration, 10903 New Hampshire Avenue, Silver Spring, Maryland 20993, United States; ‡ Nebraska Veterinary Diagnostic Center, School of Veterinary Medicine and Biomedical Sciences, 14719 University of Nebraska-Lincoln, 4040 East Campus Loop North 115Q NVDC, Lincoln, Nebraska 68583-0907, United States; § 57652 US Meat Animal Research Center, USDA Agriculture Research Service, 844 Road 313, Clay Center, Nebraska 68933, United States

**Keywords:** Moraxella bovis, infectious bovine keratoconjunctivitis, polysialic acid, chondroitin, polysaccharide structure, bacterial capsule

## Abstract

*Moraxella bovis* is a major etiologic agent for infectious bovine keratoconjunctivitis (IBK), commonly known as bovine pink eye. IBK has been a major economic burden to the cattle and dairy industries due to its economic and welfare impacts on affected cattle herds. Antimicrobial treatment of acute IBK infections is often challenging. Vaccine formulations widely used in industry have poor efficacy for the prevention of IBK. Capsular polysaccharides of some bacterial pathogens are important epidemiological markers and are successfully used in vaccines for humans. Currently, there are limited data demonstrating the presence of capsular polysaccharides in *M. bovis*. In this study, we show by genomic analysis that a broad selection of *M. bovis* strains obtained from the eyes of cattle harbor a gene cluster for expressing capsular polysaccharides. The isolates potentially express either a chondroitin-like polysaccharide or an α(2–8) polysialic acid. We isolated a polysaccharide from cultures of a well-studied model strain for IBK, the Epp63 strain, structurally identical to capsule α(2–8) polysialic acid of the human pathogens *Escherichia coli* K1 and *Neisseria meningitidis* Group B. The gene cluster in *M. bovis* Epp63 encodes a polysialyltransferase similar to other bacterial polysialyltransferases. Other *M. bovis* strains analyzed in this study possess a gene homologous to that of bacterial chondroitin synthase. We isolated a capsular polysaccharide from *M. bovis* genotypes 1 and 2 that has the repeat unit identical to nonsulfated chondroitin. These findings provide a tool for the study of *M. bovis* IBK pathogenesis that could lead to approaches for better control of the disease.

## Graphical abstract

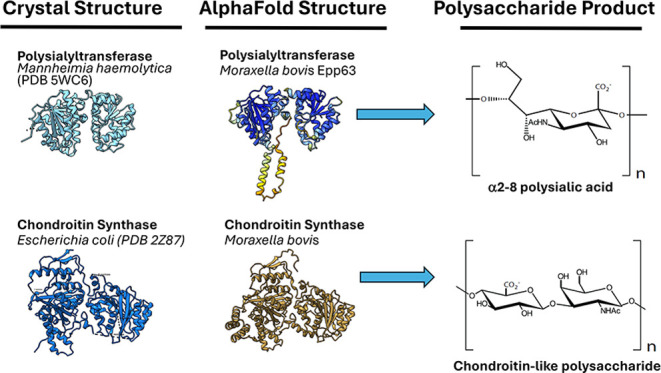

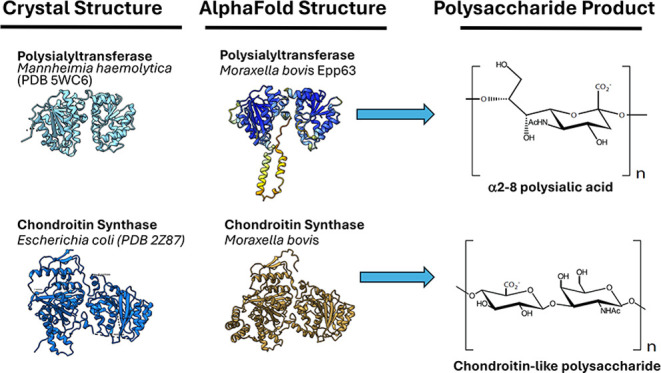

Infectious bovine keratoconjunctivitis (IBK) is the most common ocular disease in cattle worldwide. IBK is an animal welfare and economic burden for the cattle industry.^1,2^ While other bacteria have been isolated from the eyes of cattle with IBK, *Moraxella bovis* is a commonly accepted etiologic agent for this disease and is the only bacterial species that has been shown to produce IBK disease experimentally.^3^ While commercial vaccines are available for the prevention of IBK, their effectiveness at controlling the disease has not been demonstrated in the field. Autogenous vaccines have also not been shown as efficacious in the field.^4–7^ Thus, there are currently no reports of any IBK vaccine showing significant protection in the field.

While *M. bovis* is frequently isolated from the eyes of calves with IBK, it can also be isolated in the field from the eyes of healthy animals; thus, its role in virulence is complex. There are several cellular components of *M. bovis* that have been studied as virulence factors for IBK. These include type IV pili, RTX toxins, lytic enzymes, and surface carbohydrates. Indeed, pili have been included as a component of a vaccine against IBK.^3,8–10^ Two major genotypes of *M. bovis* have recently been identified that differ in their genetic makeup, although each has multiple virulence factors and has been isolated from the eyes of cattle with IBK.

The genotype 2 *M. bovis* strain Epp63 was isolated in 1963 from the surface of the eye of an IBK case. Since then, Epp63 has been used extensively for research on IBK and has been used in several clinical pathological models.^11,12^ This strain has reproducibly been shown to produce IBK under experimental conditions and was used to study several of the potential virulence factors in IBK.^13,14^ Epp63, and similar strains, are components of a commercially available vaccine to prevent IBK (Merck vaccine BOVILIS PILIGUARD PINKEYE, https://www.merck-animal-health-usa.com/retail/product-assets/cattle/piliguard-pinkeye-1-trivalent).

Recently, the whole genome sequence of Epp63 has been reported.^15^ In our effort to investigate the mechanism by which encapsulated Gram-negative pathogens synthesize their capsular polysaccharide virulence factors, we sought homologues to the enzymes that synthesize the polysialic acid capsules of *Escherichia coli* and *Neisseria meningitidis*. During our search, we found genes in the strain Epp63 genome similar to those that encode a key enzyme in the biosynthesis of α2–8 polysialic acids for the neuropathogens, *E. coli* K1 and *N. meningitidis* Group B. This polysialic acid has the same repeating structure as some mammalian host carbohydrates and is postulated to play a role in these bacteria evading host defense by mimicking self-antigens.^16^

In this report, we show that these putative capsule genes are functional in *M. bovis* and that indeed *M. bovis* Epp63 produces an α2–8 polysialic acid capsule. We also show through analysis of the genome sequences of 40 *M. bovis* strains isolated from IBK cases that genomes of these IBK isolates contain nucleotide sequences that encode production of a chondroitin-like polysaccharide, another mimic of a host cell antigen. These findings provide further data that could help elucidate the mechanism of virulence of *M. bovis* in IBK and explain the difficulty of immunization against this disease.

## Results and Discussion

### Capsule Gene Cluster of *M. bovis* Strain Epp63

The enzymes required for the biosynthesis of the polysialic acid capsule of neuropathogenic *E. coli* K1 and K92 are encoded by the genes *neuSECABD*, illustrated in Figure 1. The capsule polymerase gene, *neuS*, encodes a polysialyltransferase. In our search of gene databases for homologues of *E. coli* polysialyltransferases, we found a gene homologous to the *E. coli* *neu*S gene in *M. bovis* and *Moraxella nonliquefaciens*. Previously, a surface polysaccharide antigen of *M. nonliquefaciens* was confirmed to be α(2–8) polysialic acid, identical to the capsule of *E. coli* K1.^17^ Our analysis suggests that in addition to the *neu*S gene, *M. bovis* Epp63 also contains the genes necessary to produce a polysialic acid capsule, although not all of the genes are contiguous in Epp63 as seen in Figure 1. A multiple alignment of the *M. bovis* and *M. nonliquefaciens* NeuS proteins with those of other bacteria expressing polysialic acid capsules revealed a 33 amino acid sequence insertion (Figure 2), which suggests that the putative *Moraxella* polysialyltransferases may have a similar but modified structure.

**Figure 1:**
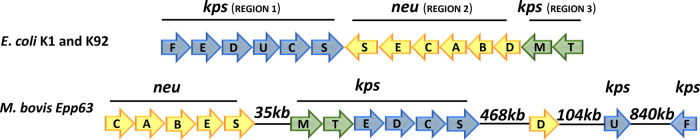
*E. coli* K1, *E. coli* K92, and *M. bovis* Epp63 gene clusters encoding polysialic acid capsule.

**Figure 2:**
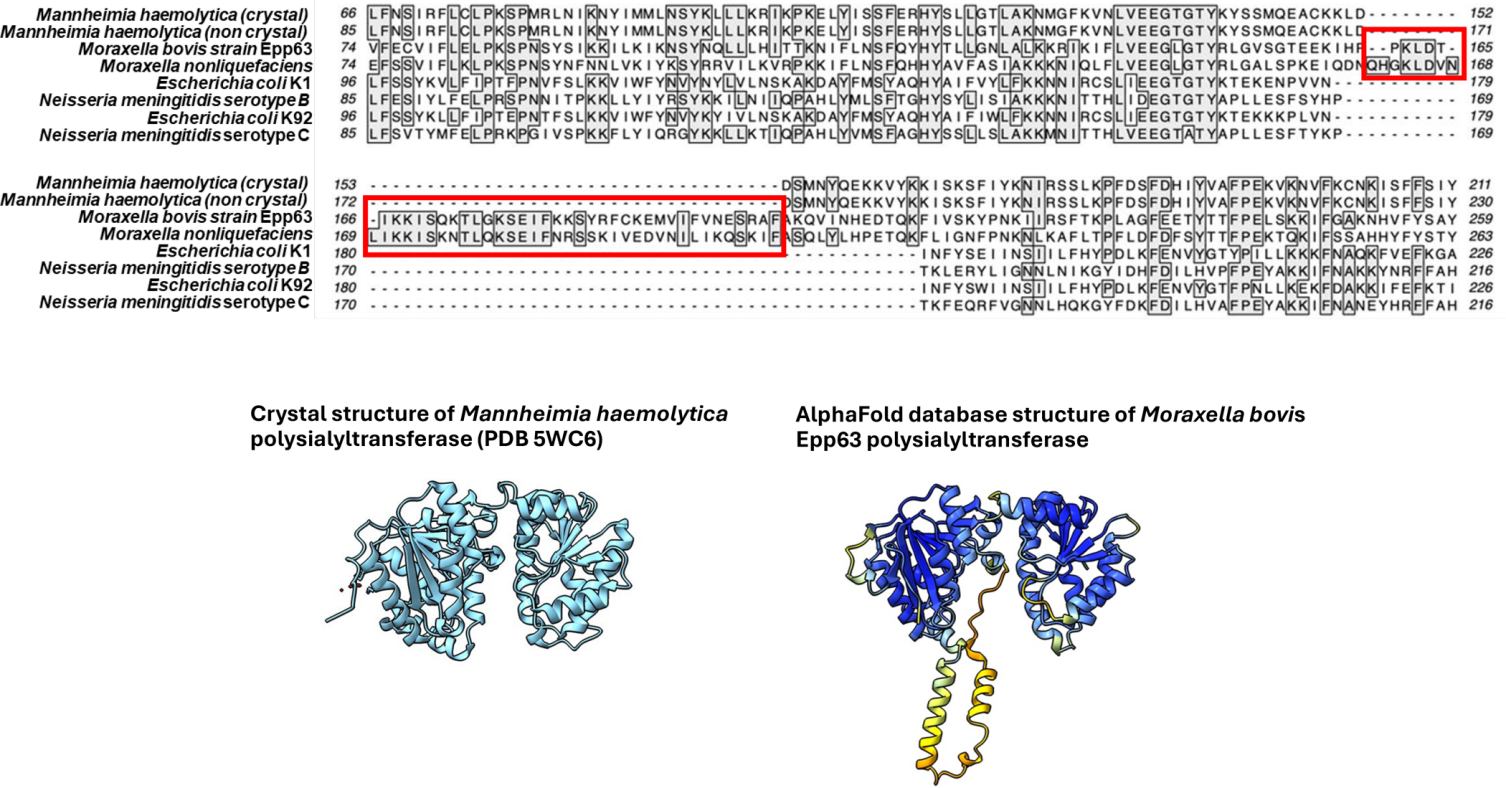
Partial bacterial polysialyltransferases sequence alignment includes the polysialyltransferase sequence of *Mannheimia haemolytica* (GenBank #QEB64866.1­(”noncrystal”)), *M. haemolytica* polysialyltransferase crystal structure (chain A, accession #5WC6, (“crystal”)), *M. bovis* Epp63 (GenBank # WP_112741710.1), *Moraxella nonliquefaciens* (GenBank # WP_239258942.1), *E. coli* K92 (GenBank# AAA24215.1), *E. coli* K1 (GenBank# AAA24213.1), *N. meningitidis* serogroup B (GenBank#QXZ29499), and *N. meningitidis* serogroup C GenBank# CAM09375.1. The intervening nonhomologous sequence unique to *M. bovis* Epp63 and/or *M. nonliquefaciens* is highlighted in the red box.

Recent reports suggested that *M. bovis* strain Epp63 does not express a capsular polysaccharide.^18^ This observation, along with the unusual sequence of the polysialyltransferase, led us to further investigate whether the observed polysialic acid gene cluster is functional and whether *M. bovis* Epp63 is indeed encapsulated.

### Capsule Expression in Strains Associated with IBK

*M. bovis* strain Epp63 was shown above to have a gene cluster characteristic of bacteria capable of expressing a polysialic acid capsule. Although the organization of the genes in *M. bovis* differs from that of the *E. coli* K1 capsule gene cluster, we found intact homologues of all of the *E. coli* genes (Figure 1). In *M. bovis* Epp63, the genes essential for polysialic acid capsule synthesis, *neu*CABES and *kps*MTEDCS, are distributed among two subclusters separated by 35.7 kb in a 47.55 kb region of the genome. Three other genes present in the *E. coli* gene cluster, neuD, kpsU, and kpsF, are present but scattered throughout the Epp63 genome. Therefore, we tested cultures of Epp63 for the detection of polysaccharides that cross-react with the antiserum that has shown specificity for the *E. coli* K1 and meningococcal group B polysialic acid capsules. As shown in Figure 3, the *M. bovis* Epp63 culture suspension gave a precipitin reaction when treated with meningococcal group B antiserum, whereas the *M. bovis* strain SAM102601, lacking the polysialic acid-specific gene cluster, did not give a precipitin reaction, suggesting the presence of α(2–8) polysialic acid only in Epp63 cultures. We isolated and purified a sialic acid-containing polysaccharide from the culture supernatant of Epp63, which we quantitated by the resorcinol colorimetric reaction^19^ and detected with MenB antiserum against α2–8 polysialic acid capsules.

**Figure 3:**
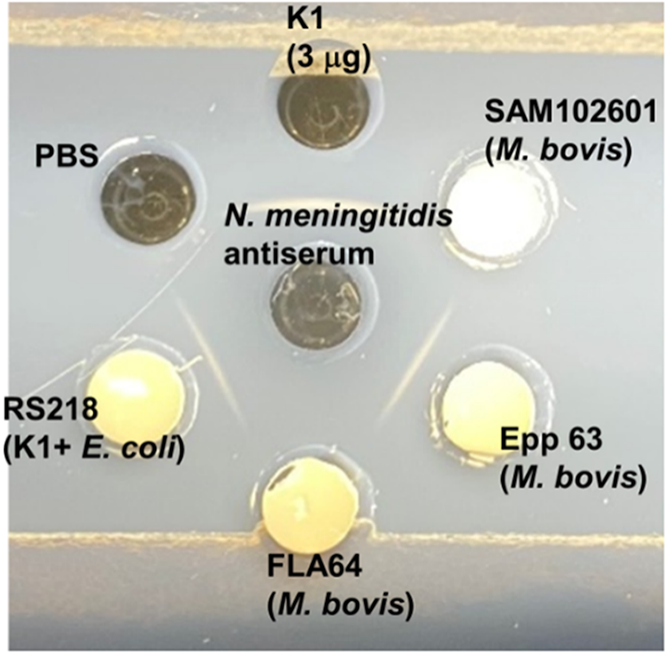
Detection of polysialic acid in *M. bovis* Epp63 cultures by immunodiffusion. Polysialic acid capsule was detected in *M. bovis* cultures by using an agar gel immunodiffusion assay. Several colonies of *M. bovis* cultured on solid media were suspended in PBS and added to one of 6 peripheral wells in the agar and diffused against meningococcal B equine antiserum in the center well for 1 h at room temperature and then at 4 °C. Wells were examined for precipitate line formation after 24 h. Negative and positive control wells contained PBS only and a 0.15 mg/mL solution of purified *E. coli* K1 capsular polysaccharide, respectively.

### Structure of Polysialic Acid in *M. bovis* Epp63

The acidic polysaccharide was isolated from cultures of *M. bovis* Epp63 by precipitation with Cetavlon detergent and purified for analysis. The ^1^H 1D NMR spectrum (not shown) of the isolated polysaccharide can be overlaid with what would be expected for a reference α(2–8) polysialic acid polysaccharide, confirming what was predicted by the serology results above. To confirm its structure, 2D NMR ^1^H, ^13^C-HSQC and long-range HSQMBC (LR-HSQMBC) NMR spectra were collected for the purified Epp63 polysaccharide and compared with the spectra of polysialic acid isolated from *E. coli* K1 (Figure S1). The spectra shown in Figure 4 have the same ^1^H,^13^C NMR signals as expected for α(2–8) polysialic acid.^20,21^ The primary structure was confirmed through long-range correlations provided by the LR-HSQCMBC experiment (shown in red in Figure 4), which enabled us to confirm the polymer glycosidic linkage between H8 of residue N and C2 of residues N + 1 (red square in Figure 4). Furthermore, the C2 assignment was possible through the cross-peaks observed between H3a and H3e to the ^13^C signal at ca. 104 ppm. This result confirms that the α(2–8)-linked polysialic acid of the *E. coli* K1 and the Epp63 polysaccharide have identical molecular structures. No signals were detected for O-acetyl groups, suggesting that the *M. bovis* polysialic acid is not O-acetylated, as has been found for several other bacterial polysialic acids.^22^

**Figure 4:**
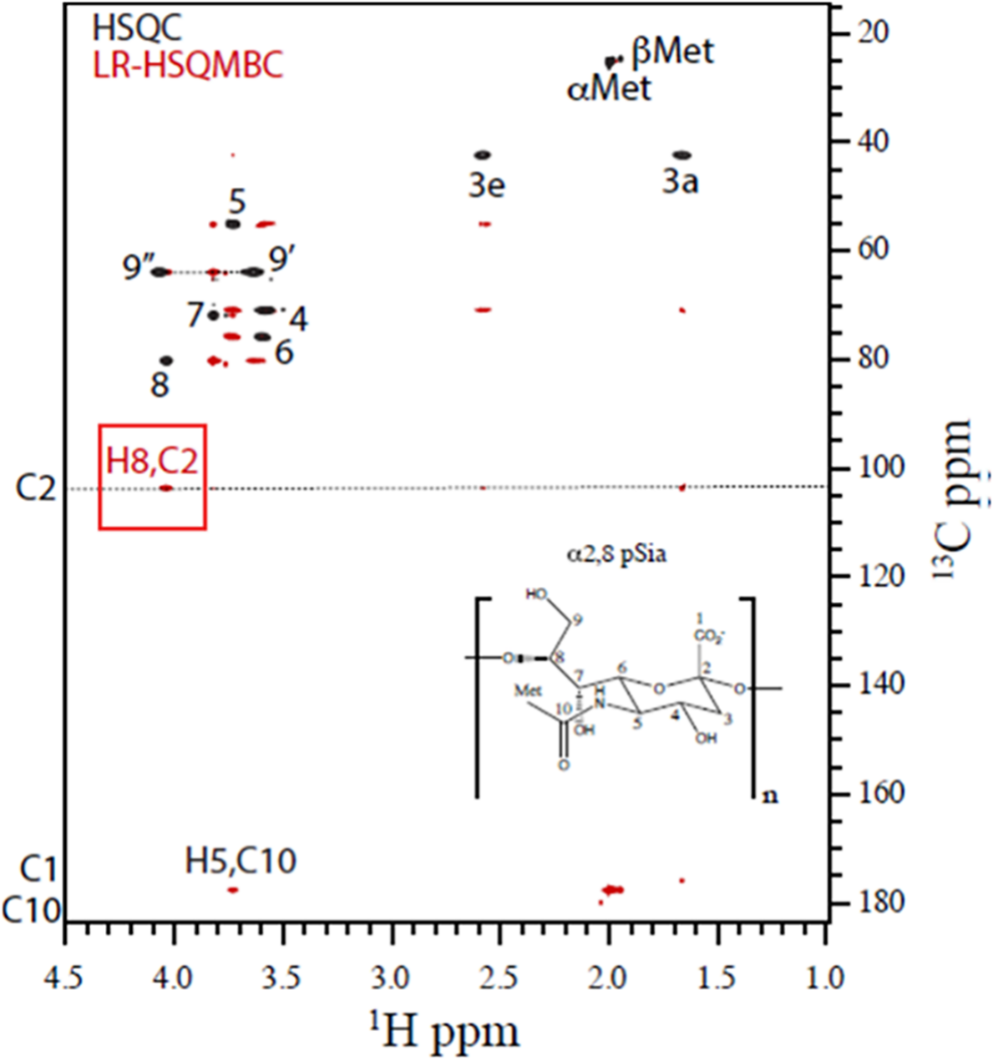
Overlaid ^1^H, ^13^C-HSQC (black) and LR-HSQMBC (red) of the purified polysaccharide from *M. bovis* Epp63 cultures. The spectra were collected at 25 °C, pH 7, on a 16 mg/mL *M. bovis* Epp63 capsular polysaccharide sample. Resonance assignments, in Arabic numerals, are consistent with those reported for α(2–8)-linked 5-N-acetylneuraminic acid polymer (α(2–8) polysialic acid or pSia, inset). The dotted line along the C2 frequency is included to serve as a guide for all ^1^H atoms that are long-range scalar-coupled to C2. The red square highlights the H8,C2 correlation detected in the LR-HSQMBC experiment, confirming the α(2–8) glycosidic linkage.

### Expression of *M. bovis* Epp63 Polysialyltransferase in *E. coli*

We expressed the *M. bovis* polysialyltransferase gene, *neuS* in *E. coli*, to test its function as it is predicted by the AlphaFold Protein Structure Database of sequence-based 3D model structures to have a slightly different protein structure compared to the only published crystal structure of a bacterial polysialyltransferase, that of *M. haemolytica* serotype A2.^23^ The polysialyltransferase of *M. bovis* Epp63 was purified and assayed for its ability to elongate polysialic acid chains in the presence of the expected donor substrate CMP-sialic acid. The enzyme appears to prefer α(2–8) polysialic acid acceptors and exhibited little activity when α(2–9) polysialic acid was added as an acceptor (data not shown). This observation is consistent with the hypothesis that *M. bovis* neuS encodes an α(2–8) polysialyltransferase.

We used a fluorescence-based HPLC assay^24^ to compare the ability of meningococcal Group C and *M. bovis* Epp63 polysialyltransferase to use two donor substrates, CMP-*N*-acetylneuraminic acid and CMP-*N*-glycolylneuraminic acid (Figure 5). Cattle express *N*-glycolylneuraminic acid on their glycoproteins, whereas humans do not.^25,26^ Nevertheless, our results suggest no detectable difference in the donor substrate of the *Neisseria* polysialyltransferase and that of the bovine pathogen, *M. bovis* Epp63. A high molecular weight polysialic acid peak was observed after incubation of either enzyme for 30 min with CMP-sialic acid. In contrast, only low molecular weight oligosaccharides were observed after a 20 h incubation period of either enzyme in a reaction mixture containing CMP-NGc as the donor substrate.

**Figure 5:**
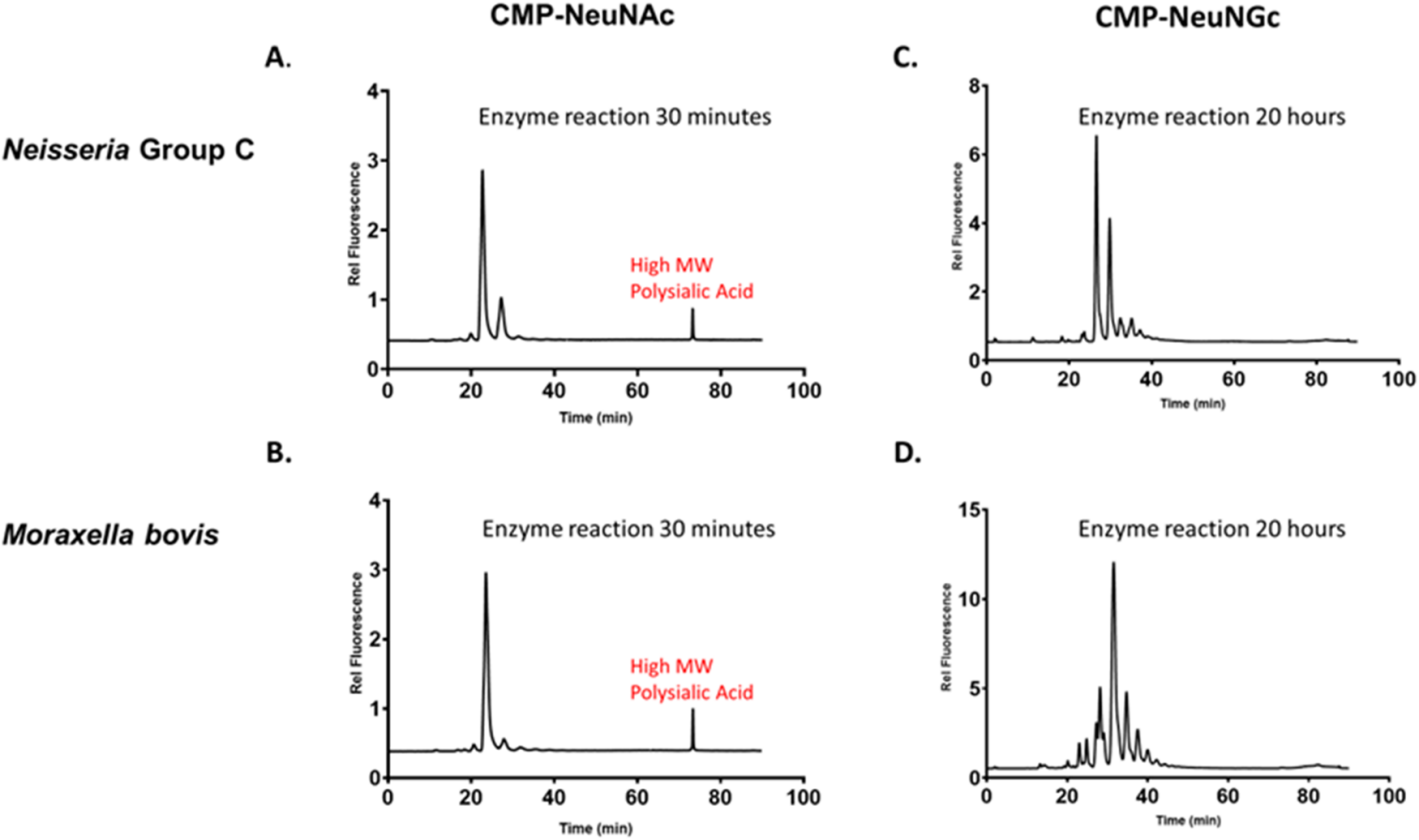
Comparison of the activity of purified *M. bovis* Epp63 polysialyltransferase with *N. meningitidis* Group C polysialyltransferase. *M. bovis* Epp63 and *N. meningitidis* Group C polysialyltransferases (PST) were assayed for polymerase activity by HPLC ion exchange chromatography using the fluorescent acceptor substrate GD_3_FCHASE. (A) *N. meningitidis* PST and (B) *M. bovis* PST were incubated with CMP-N-acetylneuraminic acid for 30 min prior to chromatography. Similarly, the PST enzymes were incubated with CMP-*N*-glycolylneuraminic acid (C) *N. meningitidis* and (D) *M. bovis* for 20 h due to the slow reaction rate with this acceptor.

### Analysis of Capsule Gene Clusters of Other *M. bovis* Strains Associated with IBK

The genes in *E. coli* K1 and K92 required for the synthesis of the specific capsular polysaccharide polysialic acid are *neu*A (CMP-sialic acid synthetase), *neu*C (sialic acid synthase), and *neu*S (polysialyltransferase). An analysis of our database of over 200 genome sequences from IBK isolates of *M. bovis* revealed that Epp63 is the only strain in the database of IBK isolates possessing the necessary genes for the production of a polysialic acid capsule. This observation poses the question of whether other strains of *M. bovis* isolated from IBK lesions harbor genes that support capsular polysaccharide synthesis. To address this question, we searched the database for genes necessary for the production of other ABC transporter-dependent capsules, namely, *kps*MT and *kps*CS. An alignment was generated with over 40 *M. bovis* gene clusters that possessed sequences homologous with *kps*MT and *kps*CS. The alignment of the putative KpsC protein sequences in *M. bovis* and *M. bovoculi* isolates with *E. coli* KpsC is shown in Figure S2. Further analysis of these 40 *M. bovis* gene sequences also revealed the presence of genes encoding a chondroitin-like capsular polysaccharide polymerase (Figure S3) homologous with the *E. coli* K4 enzyme. All of the *M. bovis* genome sequences, except strain Epp63, possessed capsule-related gene cluster sequences homologous with *E. coli* K4. Similarly, the genome of *M. bovoculi* genotype 2 isolates had sequences that were homologous to *E. coli* K4 capsule gene sequences.^27^ The *E. coli* K4 capsule is a nonsulfated chondroitin-like polysaccharide with a disaccharide repeat unit: (1–3)-β-N-acetyl-galactosamine and (1–4)-β-glucuronic acid. The discovery of the capsular polysaccharide gene clusters described in this report in isolates from IBK cases implies that *M. bovis* has primarily 2 types of capsular polysaccharides, either nonsulfated chondroitin or polysialic acid. The organization of gene clusters homologous to the *E. coli* K4 capsule cluster is illustrated in Figure 6. While all of the genes found in *E. coli* K4 related to capsular polysaccharide production are present in the *M. bovis* genomes, they are organized in subclusters spaced as was described above for *M. bovis* Epp63 (Figure 1).

**Figure 6:**
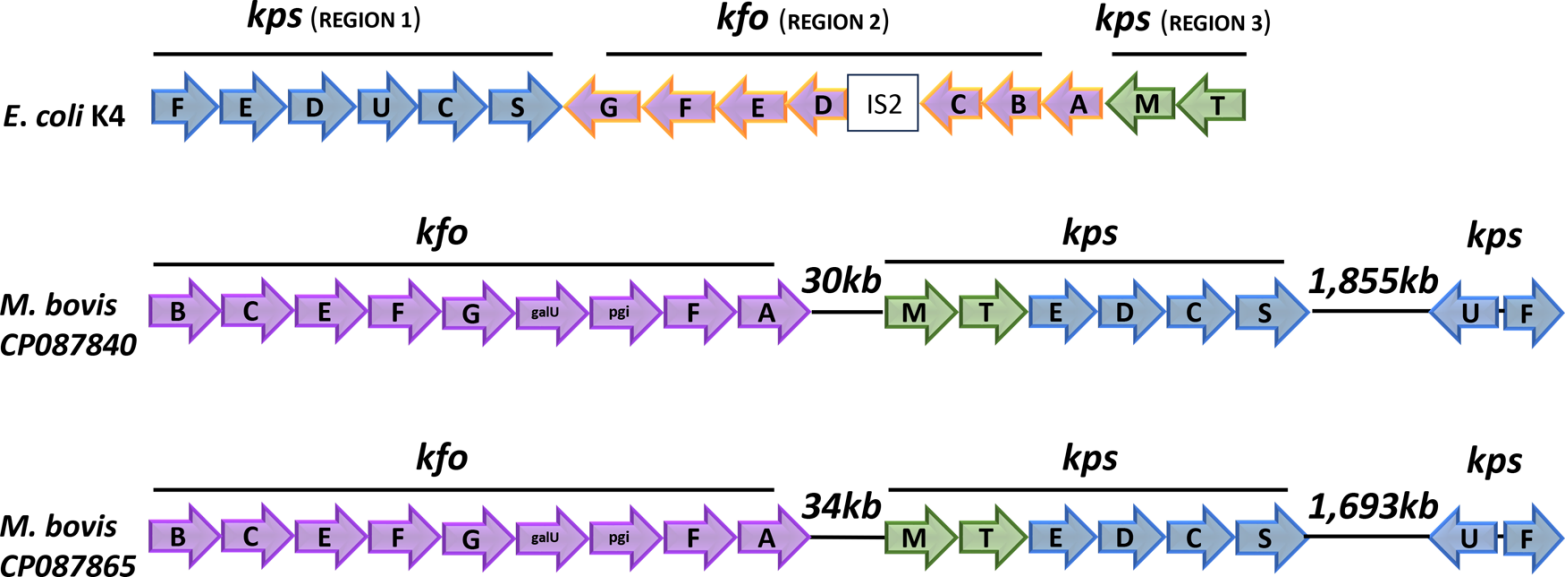
*E. coli* and *M. bovis* gene clusters encoding chondroitin capsules.

We performed multiple alignments of the amino acid sequences of putative *M. bovis* chondroitin synthase with the sequences of bacterial chondroitin synthetases that have been previously characterized (Figure 7). Based on this alignment, the *M. bovis* chondroitin synthase is most closely related to that of the *Pasteurella multocida* enzyme. Several putative catalytic residues have been identified in the crystal structure of *E. coli* K4 chondroitin synthase. As shown in Figure 7, the putative catalytic residues are conserved in all of the *M. bovis* sequences that were analyzed, supporting our hypothesis that *M. bovis* strains produce a chondroitin-like capsule.

**Figure 7:**
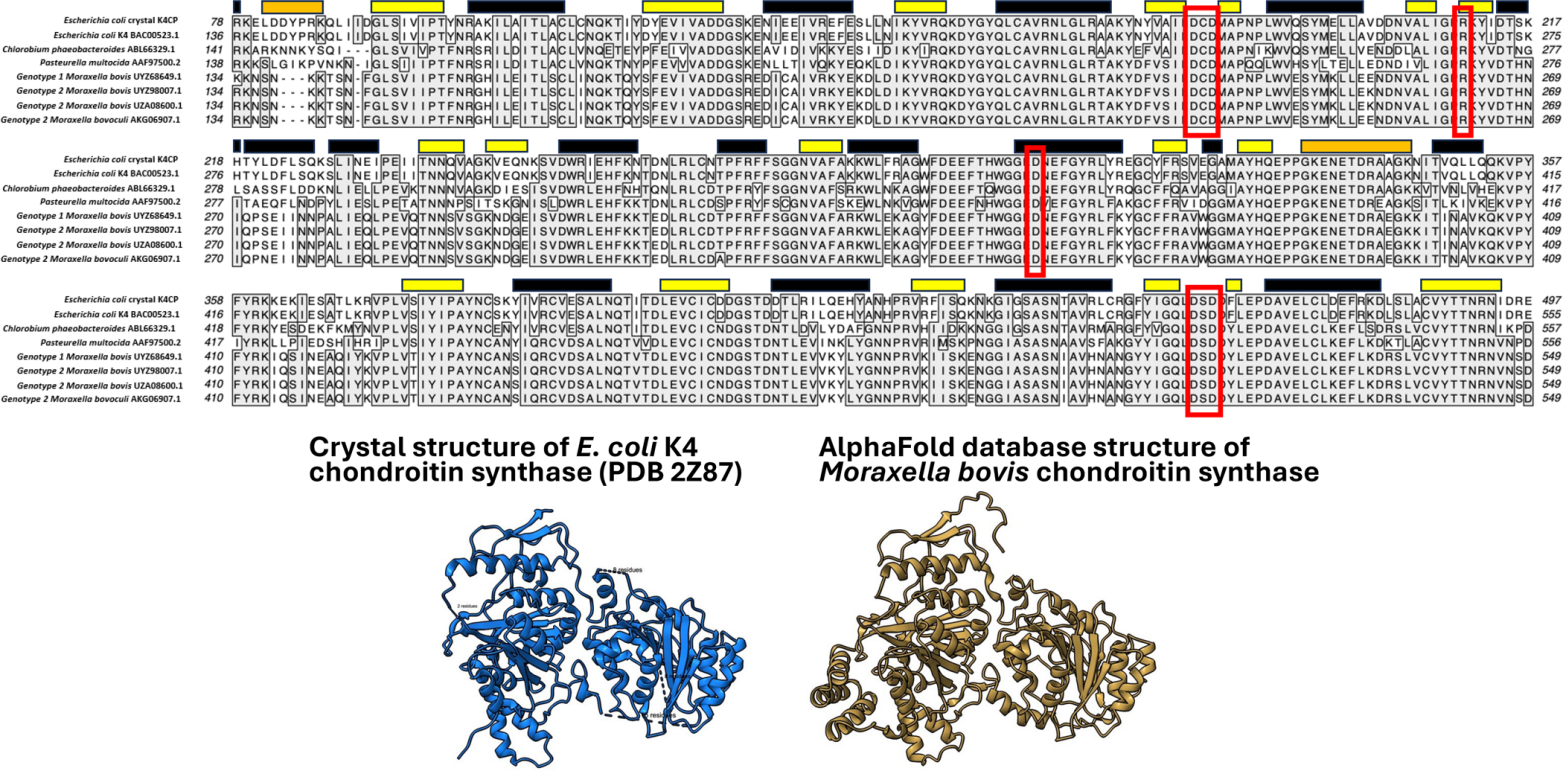
Multiple alignment of bacterial chondroitin synthase sequences. Labels in the alignment show GenBank accession numbers for the sequences. The alignment contains the chondroitin synthase sequence from the crystallized *E. coli* chondroitin synthase (K4CP). Active site residues are outlined in red. Sheet and helix motifs of the crystal structure are represented with yellow and black blocks, respectively. Unmodeled residues in the crystal structure are represented with orange blocks.

### Isolation of Capsular Polysaccharides in Other *M. bovis* Strains

Since the sequence of the gene cluster in both genotypes of *M. bovis* and genotype 2 *M. bovoculi* strains suggested the production of a chondroitin-like polysaccharide, we tested cultures for a chondroitinase-sensitive polysaccharide. Extracts of *M. bovis* genotype 1 and 2 and *M. bovoculi* genotype 2 were analyzed by agarose electrophoresis for a negatively charged polysaccharide. A chondroitinase-sensitive polysaccharide was detected in the extracts of *M. bovis* regardless of genotype, but not in the *M. bovoculi* genotype 2 strain (Figure 8).

**Figure 8:**
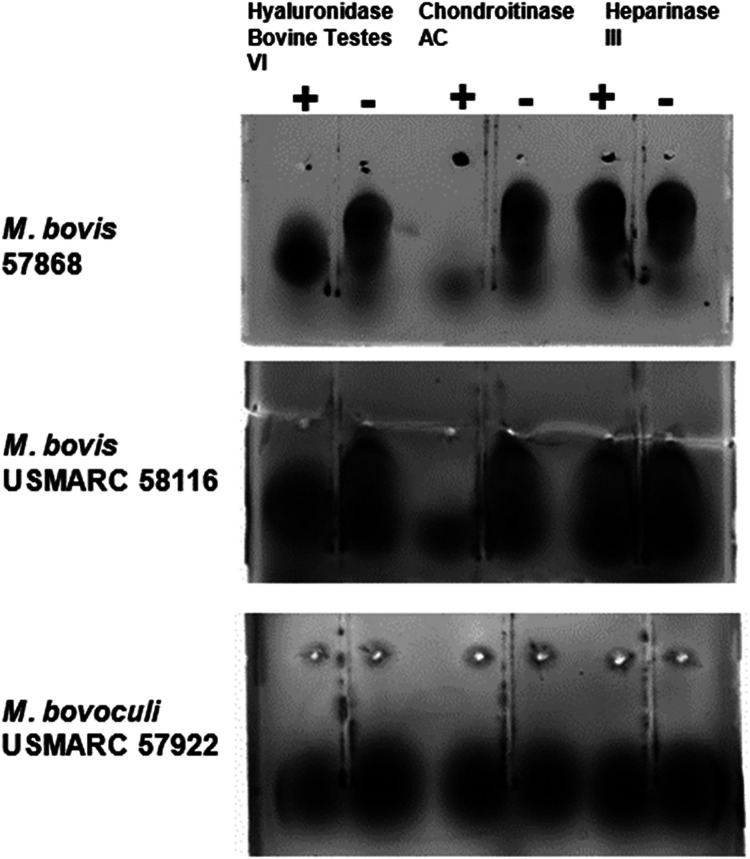
Digestion of *M. bovis* polysaccharides with chondroitinase. Polysaccharide was purified from *M. bovis* strains 57868, USMARC 58166, and *M. bovoculi* strain USMARC 57922. Polysaccharide from each strain was digested for 1 h at 37 °C with either hyaluronidase, chondroitinase AC, or heparinase III. The resulting reaction mixtures were subjected to electrophoresis in agarose gel for 2 h and stained overnight with Stains-All to visualize polysaccharides.

### Structure of *M. bovis* Chondroitin-like Polysaccharide

Polysaccharides were isolated from *M. bovis* genotype 1 and genotype 2 cultures by Cetavlon precipitation and purified as described in the Materials and Methods below. The presence of polysaccharide was monitored during purification with the carbazole uronic acid assay.^28^ *M. bovoculi* strain 57922, which has a truncated chondroitin polymerase gene, did not produce detectable polysaccharides using this method. Analysis of the NMR spectra in Figure 9 suggested that polysaccharides from both genotype 1 and genotype 2 strains are identical and consist of an unsubstituted disaccharide repeat unit of (1–4)­βGlcA(1–3)­βGalNAc. The structures of the capsular polysaccharides of *M. bovis* genotype 1 and genotype 2 were confirmed by comparing the resulting NMR spectra with those published for bacterial chondroitin-like polysaccharides, namely, defructosylated *E. coli* K4 polysaccharide and the polysaccharide previously reported for *M. bovis* strain Mb25 (Table 1).

**Figure 9:**
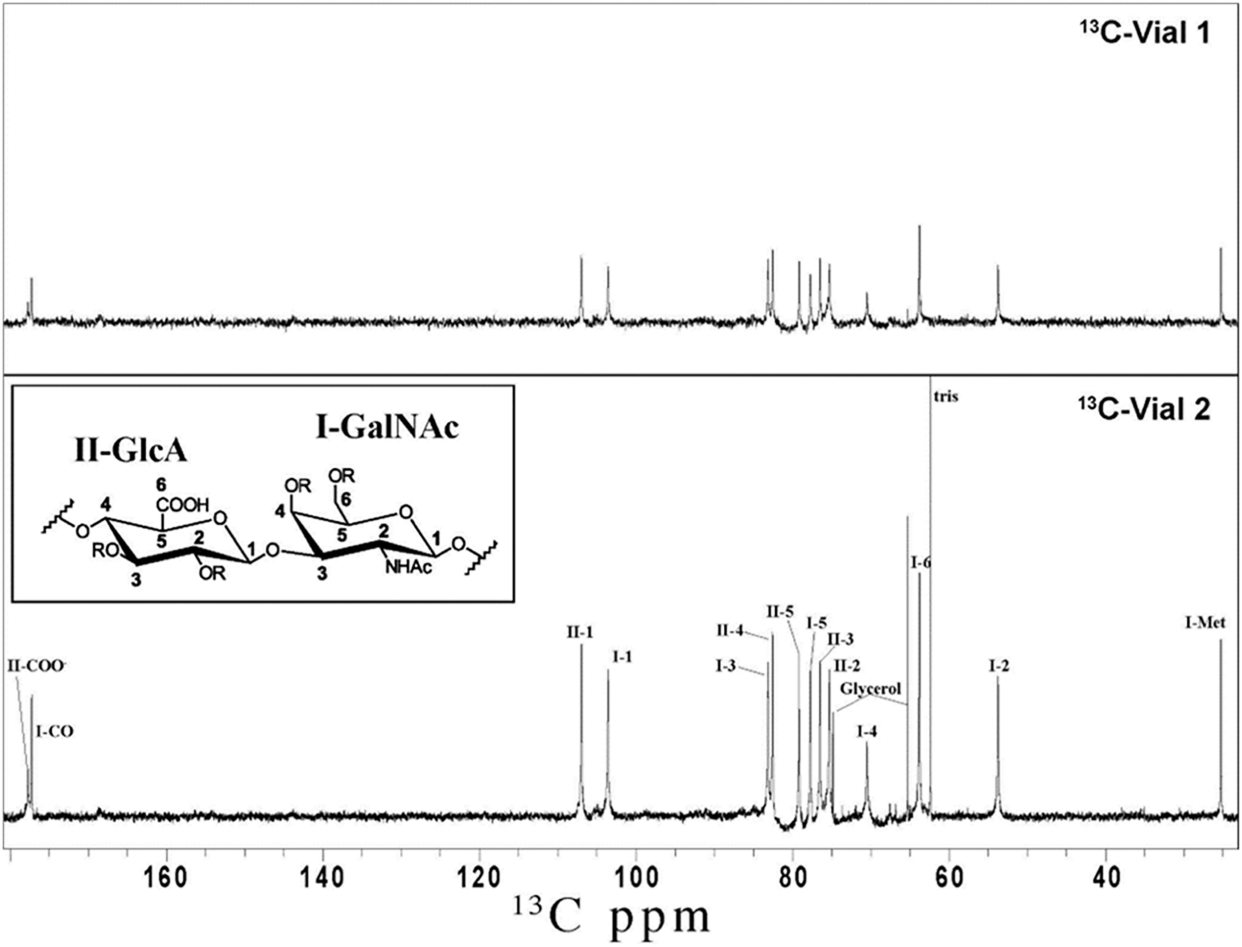
^13^C 1D NMR spectra of purified polysaccharides of *M. bovis* genotypes 1 (top panel) and 2 (bottom panel). Resonance assignments are shown in Arabic numerals for atom assignment and Roman numerals for residue assignment.

**Table 1:** Assigned Resonances (in ppm) for *M. bovis* Samples from Data Collected at 25 and 37 °C Compared to Published Literature Values

		37 °C		25 °C		25 °C-Ichikawa et al.^a^		25 °C-Wilson et al.^a^	
residue	atom number	^1^H	^13^C	^1^H	^13^C	^1^H	^13^C	^1^H	^13^C
I-GalNAc	1	4.51	103.5	4.49	103.5	4.48	103.4	4.5	104.0
	2	3.98	53.7	3.98	53.7	3.98	53.7	3.99	53.8
	3	3.79	83.1	3.79	83.1	3.77	83.2	3.84	82.8
	4	4.10	70.5	4.10	70.5	4.10	70.5	4.09	70.4
	5	3.68	77.7	3.68	77.7	3.65	77.7	3.7	77.7
	6	3.77	63.8	3.77	63.8	3.76	63.8	3.69	63.6
	Me	2.01	25.2	2.01	25.2	2.04	25.1	1.99	25.1
	CO	NA	177.8	NA	177.7	NA	177.6	NA	177.7
II-GlcA	1	4.47	106.9	4.47	107.0	4.48	107.1	4.56	107.1
	2	3.34	75.2	3.34	75.2	3.34	75.2	3.38	74.8
	3	3.56	76.4	3.56	76.4	3.56	76.4	3.66	76.4
	4	3.74	82.5	3.73	82.5	3.74	82.5	3.82	82.7
	5	3.67	79.1	3.67	79.1	3.67	79.1	3.99	76.4
	COO-	NA	177.4	NA	177.3	NA	177.3	NA	174.5

a Doi: 10.1002/mrc.5426 and Doi: 10.1016/j.carres.2004.12.016.

## Methods

### Strains

The following strains were cultured and used in this study (Table S1): Genotype 1 *M. bovis* strain 57868 (NCBI accession # CP087865), genotype 2 *M. bovis* strain Epp63 (NCBI accession # CP030241), genotype 2 *M. bovis* strain 58116 (NCBI accession # CP087840),^29^ genotype 2 *M. bovis* 102601 (NCBI accession # CP087825), genotype 1 *M. bovoculi* strain 57922 (NCBI accession # CP011380),^30^ and genotype 2 *M. bovoculi* strain 22581 NCBI accession # CP011376. The strains were passaged twice on fresh 12.5% Blood Agar plates and incubated for 18 h at 37 °C prior to use.

### Expression of *M. bovis* NeuS in *E. coli*

The *M. bovis* neuS gene was expressed in *E. coli* using 2 expression plasmids, pET151-NeuS and pMAL c6T-NeuS (synthesized by Thermo Scientific GeneArt), based on the nucleotide sequence obtained for strain Epp63. Plasmid pET151-NeuS was transformed into *E. coli* BL21­(DE3). Fresh transformants on an LB ampicillin plate resuspended in 5 mL of media were used to inoculate 1L of 2X YT broth containing 100 μg/mL ampicillin. The culture was shaken at 150 rpm, 37 °C until OD 600 nm = 0.6 to 0.8. Approximately 0.13 g of IPTG in 500 μL of H_2_O was added to each culture with shaking at 120 rpm, 20 °C, 21–22 h. Cells were harvested by centrifugation at 6500*g*, 30 min, and 2–8 °C.

### Purification of Polysialyltransferase

Plasmid pMAL c6T-NeuS was transformed into *E. coli* Arctic Express cells (Agilent 230191) and grown in 1 L of 2X YT medium as described above. The harvested cell paste (10.23g) was resuspended in 25 mL Roche cOmplete EDTA-free Protease Inhibitor Cocktail (04693132001) and approximately 10 mg PMSF (Sigma 1135906100), lysed in a French pressure cell, and then centrifuged at 39,000*g* × 90 min, 2–8 °C. The supernatant was tumbled overnight with 5 mL amylose resin at 2–8 °C, which had been equilibrated in 20 mM Tris–HCl, 1 mM EDTA, and 200 mM NaCl, pH 7.5 (column buffer). The suspension was packed onto a column and washed with 20 column volumes of a column buffer. The enzyme was eluted with 5 × 10 mL of 100 mM amylose in column buffer. Elution fractions containing the enzyme were adjusted to 10% glycerol and stored at −80 °C.

### Assay of Polysialyltransferase Activity

Polysialyltransferase activity was assayed by paper chromatography as described previously^31^ using colominic acid as the acceptor substrate or by HPLC using a fluorescence-labeled GD3 analogue as the acceptor substrate.^24^

### Isolation of *M. bovis* and *M. bovoculi* Capsular Polysaccharides

A single colony of *M. bovis* and *M. bovoculi* strains was used to inoculate a preinoculum and grown 20 h at 37 °C with shaking in Minimal Davis Media supplemented with dextrose (1 g/L), 100 mL of LMW (10×) yeast extract per liter, CaCl_2_ (1 mM), Thiamine (20ug/L), and Heme (1uM). The preculture was used to inoculate 1.5 L of the same medium and shaken for 22–26 h at 37 °C. Strain Epp63 was cultured on the same media without the addition of CaCl_2_, Thiamine, and Heme.

Capsular polysaccharide was isolated and purified according to the procedure described by Willie F. Vann and Stephen Freese,^32^ with some modifications described as follows. The acidic capsular polysaccharide and the bacterial cells were precipitated from the liquid cultures by the addition of an equal volume of 0.2 hexadecyltrimethylammonium bromide (Cetavlon) and stored for 48 h at 4 °C, followed by centrifugation (16,000*g* × 30 min). The Cetavlon cell paste was stored overnight at −80 °C, resuspended in 50 mL 1 M calcium chloride to extract polysaccharide from the precipitate, and followed by homogenization and centrifugation (16,000*g* × 30 min). The supernatant was adjusted to 25% ethanol, stirred, and then centrifuged (12,000*g* × 30 min) to remove cell debris, precipitated nucleic acid, and protein. The supernatant was adjusted to 80% ethanol, followed by centrifugation to isolate the crude polysaccharide fraction. The paste was dissolved in 10% saturated sodium acetate (pH 7.5), followed by an equal volume of cold buffered phenol and shaken vigorously for 30 min in an ice bath. The phases were separated by centrifugation (12,000*g* × 30 min). The upper aqueous phase was removed and adjusted to 25% ethanol, stored overnight at 4 °C, and then centrifuged (12,000*g* × 30 min). The polysaccharide was recovered after an additional 2 cycles of phenol extraction and ethanol precipitation. The final 80% ethanol precipitate was dissolved in water and dialyzed extensively against water before being lyophilized. The glucuronic acid content of the recovered polysaccharide was determined using the carbazole method.^28^

### Immunological Assays

Polysialic acid capsule was detected by using an agar gel immunodiffusion assay (AGID). Seven wells, including a center well and six peripheral wells, were cut into a 0.9% type II agarose gel solution. The center well received 20 μL of antimeningococcal B whole serum of equine origin (H46)^33^ before the gel was incubated for 1 h at room temperature. Several colonies of the *M. bovis* strains to be examined were suspended in PBS, and 20 μL of the resulting suspension was added to one of 6 peripheral wells in the agar. Control wells included equal volumes of PBS only and a 0.15 mg/mL solution of purified *E. coli* K1 capsular protein. The agarose gel was incubated at 4 °C and examined for precipitate line formation after 24 h.

### Sensitivity of Polysaccharides to Digestion

Capsules produced by strains possessing chondroitin genes were digested according to Wu et al.^34^ with minor modifications. Each purified capsule polysaccharide (20 μg) was digested at 37 °C for 1 h with either 1U of Hyaluronidase (Sigma H3757) in 20 mM MOPS, pH 5.4, 0.01U of Chondroitinase AC (Sigma C2780) in 50 mM Tris pH 7.5, or 0.02U of Heparinase III (Sigma H8891) in 50 mM Tris pH 7.5. Digested polysaccharide (10 μL) was loaded on a 0.5% agarose gel and electrophoresed at 50 V and room temperature for 2 h. Gels were stained overnight with 0.005% Stains-All (Sigma E-9379) in 50% ethanol.^35^

### Analysis of Polysaccharide Structures by NMR

#### Sample Preparation

The lyophilized polysaccharide was dissolved in 500 μL of ^2^H_2_O (Millipore Sigma, Burlington, Massachusetts) to a final concentration of 16 mg/mL, pH 7. The solution contained 10 mM sodium 2,2-dimethyl- 2-silapentane-5-sulfonate-D6 (DSS) as an internal reference for NMR signals.

#### Data Collection and Processing

NMR spectra were acquired at 25 °C, on a Bruker Avance III 700 MHz spectrometer equipped with an xyz-gradient triple resonance TCI CryoProbe, using Topspin 3.6.5 (Bruker, Billerica, MA). The spectra were processed with Topspin 4.1.1 (Bruker, Billerica, MA). The ^1^H, ^13^C-HSQC (Bruker sequence hsqcetgp) and long-range HSQMBC^36^ were collected using the parameters presented in Table 2.

**Table 2:** NMR Experiments Acquisition Parameters Used to Collect the Data Shown in Figure 4

	^1^H, ^13^C-HSQC		LR-HSQMBC	
acquisition parameter				
dimension	^1^H	^13^C	^1^H	^13^C
spectral window (ppm)	10	100	3	200
complex points	2048	256	3000	512
resolution after zero-filling (Hz/point)	3.5	69	1.7	69
carrier (ppm)	4.7	60	4.7	100
scans/t1 point	32		256	
recycle delay (s)	1.7		1.8	
*J*_CH_ for INEPT transfer (Hz)	145		20	

The spectra were processed in Topspin 4.0 and analyzed with CCPNmr Analysis 2.4.^37^ The free induction decays (FIDs) for the direct and indirect dimensions were zero-filled twice their size and subsequently apodized using squared cosine-bell functions to yield spectra with the resolution presented in Table 2. The ^1^H frequencies were referenced to the internal DSS ^1^H signal and the ^13^C frequencies were referenced indirectly from the ^1^H DSS reference frequency.^38^

#### Resonances Assignment

For α(2–8) polysialic acid assignment, we used a combination of ^1^H 1D, ^1^H, ^13^C-HSQC, and long-range (LR)-HSQMBC (Bruker sequences zgpr, hsqcetgp, and hsqcetgpipjcsp2_lrlp2, respectively). All spectra were acquired using pulse sequences from the Bruker pulse sequence library with minor modifications to filter out water signals through application of *xyz* gradients between ^1^H and ^13^C 90̊ pulses in the forward and back INEPT.

For the samples containing the isolated chondroitin-like polysaccharide from *M. bovis*, a combination of 1D ^13^C and 2D ^1^H, ^13^C-HSQC, ^1^H, ^13^C-HSQC-TOCSY, ^1^H, ^13^C-HSQC-NOESY, and long-range (LR)-HSQMBC (Bruker sequences zgdc, hsqcedetgp, hsqcdietgpsi, hsqcetf3gpnofb, and hsqcetgpipjcsp2_lrlp2, respectively) was used. All spectra were acquired using pulse sequences from the Bruker’s pulse sequence library (when applicable, we introduced a minor modification to filter out water signals through application of *XYZ* gradients between ^1^H and ^13^C 90° pulses in the forward and back INEPT). We collected the experiments at both 25 and 37 °C. We collected experiments at 25 °C to enable a direct comparison with available literature values. However, at 25 °C, the viscous polysaccharide sample yields somewhat broad NMR signals. Therefore, we also collected experiments at 37 °C to confirm resonance assignments. At higher temperatures, molecules tumble faster, which translates into narrower NMR signals, increased signal resolution, and improved sensitivity, thus improving the overall data quality.

For the purified polysaccharide from *M. bovis* Epp63, the experimental acquisition parameters are presented in Table 2.

### Bioinformatics Data Analysis

The Core and Pan genomes of a diverse population of *M. bovis* have previously been identified.^29^ From this data set, BLAST was used to identify genes of interest from across the population and make multiple sequence alignments.

## Supplementary Material



## Notes

Strains and plasmids used in this study (Table S1); overlaid ^1^H, ^13^C-HSQC spectra for α(2–8) polysialic acid (pSia) from *E. coli* K1 reference samples with and without Ca^2+^ (blue and red, respectively) and for *M. bovis* polysaccharide (PS) in purple, collected at 25 °C (Figure S1); multiple alignment of *M. bovis* and *M. bovoculi* sequences with the protein sequence of *E. coli* K1 KpsC (Figure S2); multiple alignment of *M. bovis* and *M. bovoculi* sequences with protein sequence of *E. coli* K4 chondroitin synthase (Figure S3) (PDF)
