# Supplementary material for: Identification of Polysialic Acid and Chondroitin-like Polysaccharides of Moraxella bovis Strains Associated with Infectious Bovine Keratoconjunctivitis
Source: ACS Infect Dis. 2025 Dec 4;12(1):152–61. doi: 10.1021/acsinfecdis.5c00628 (PMC12797221; doi:10.1021/acsinfecdis.5c00628)

SUPPORTING INFORMATION

Identification of Polysialic Acid and Chondroitin-like Polysaccharides of *Moraxella bovis* Strains Associated with Infectious Bovine Keratoconjunctivitis

Justine Vionnet[1*], Dwight C. Peterson[1], John Dustin Loy[2], Emily Wynn[3], Marcos Daniel Battistel[1], Matthew Hille[2], Michael L. Clawson[3], Willie Vann[1*]

[1]Center for Biologics Evaluation and Research, Food and Drug Administration, 10903 New Hampshire Ave., Silver Spring, Maryland, 20993, USA

[2] Nebraska Veterinary Diagnostic Center, School of Veterinary Medicine and Biomedical Sciences, University of Nebraska-Lincoln, 4040 East Campus Loop North 115Q NVDC, Lincoln, NE 68583-0907, USA

[3] US Meat Animal Research Center, USDA Agriculture Research Service, Clay Center, 844 Road 313, Clay Center, NE 68933, USA

Corresponding authors:

E-mail address: justine.vionnet@fda.hhs.gov and Wvannsialic@outlook.com

Table of Contents

1. Strains and Plasmids used in this study……………………………………………...Table S1
2. Overlaid ^{1}H, ^{13}C HSQC spectra for α2,8 polysialic acid (pSia)………………………Figure S1
3. Multiple alignment of *M. bovis* and *M. bovoculi* sequences with the protein sequence of *E. coli* K1 KpsC……………………………………………………………………...Figure S2
4. Multiple alignment of *M. bovis* and *M. bovoculi* sequences with protein sequence of *E. coli* K4 chondroitin synthase……………………………………………………….Figure S3

Table S1. Strains and plasmids used in this study.

Strain or Plasmid	Description	Source or Reference
Bacterial Strains		
Moraxella bovis Epp63	M. Bovis strain isolated from an IBK case in 1963	This Study<br>G. W. Pugh, Jr. (National Animal Disease Center, USDA-ARS, Ames, IA, USA)
Moraxella bovis 57868		This study<br>J. D. Loy, Veterinary Diagnostic Center, University of Nebraska–Lincoln, Lincoln, NE
Moraxella bovis USMARC 58116		
Moraxella bovoculi USMARC 57922	IBK eye isolate collected in Kansas USA co infects with *M bovis*, hemolytic	This Study<br>M. Clawson<br>U.S. Dept. of Agriculture<br>U.S. Meat Animal Research Center<br>Clay Center, NE 68933
Moraxella bovoculi USMARC 22581	IBK asymptomatic nasopharyngeal *M. bovoculi* isolate collected in Missouri USA, non-hemolytic	
Escherichia coli K1	K1 capsule is a homopolymer of α−2,8-linked N-acetylneuraminic acid linked to BSI, neonatal meningitis	W. Vann
Escherichia coli K92	*Escherichia coli* Bos12 Type II capsule	W. Vann
Escherichia coli RS218	*Escherichia coli* RS218 (O18:H7:K1, ST95) Human-newborn meningitis prototype strain of neonatal meningitis	R. Silver
Plasmids		
pET151-NeuS		This Study<br>GeneArt Thermo Scientific
pMAL c6T-NeuS		This Study<br>GeneArt Thermo Scientific

Figure S1. Overlaid ^{1}H, ^{13}C HSQC spectra for α2,8 polysialic acid (pSia) from *E. coli* K1 reference samples with and without Ca^{2+} (blue and red, respectively) and for *M. bovis* polysaccharide (PS) in purple, collected at 25 ˚C.

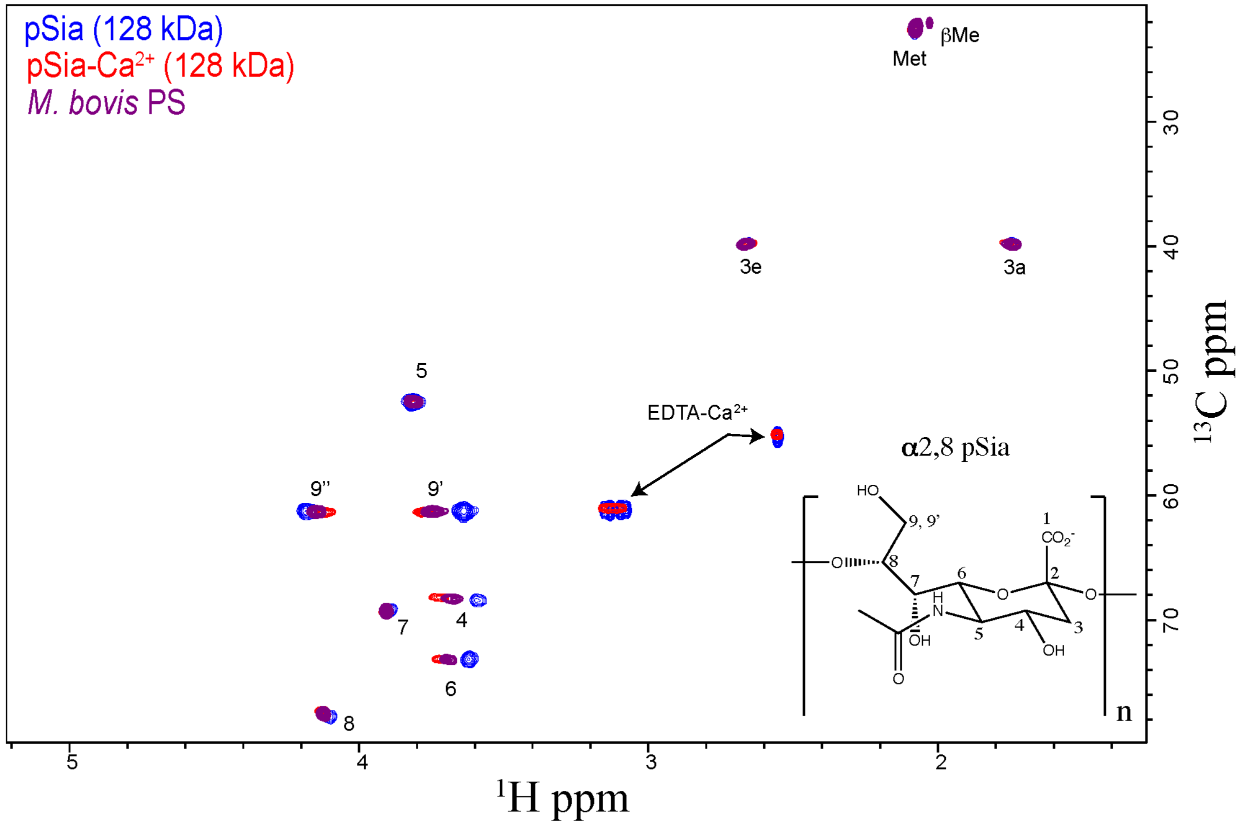

Figure S2. Multiple alignment of *M. bovis* and *M. bovoculi* sequences with the protein sequence of *E. coli* K1 KpsC.

Whole genome accession numbers for the strains from which the kpsC sequences were extracted are shown in the labels. The alignment contains partial kpsC protein sequence from the N-terminal domain of a crystalized *E. coli* kpsC. Sheet and helix motifs of the crystal structure are represented with yellow and black blocks, respectively. Unmodeled residues in the crystal structure are represented with orange blocks.

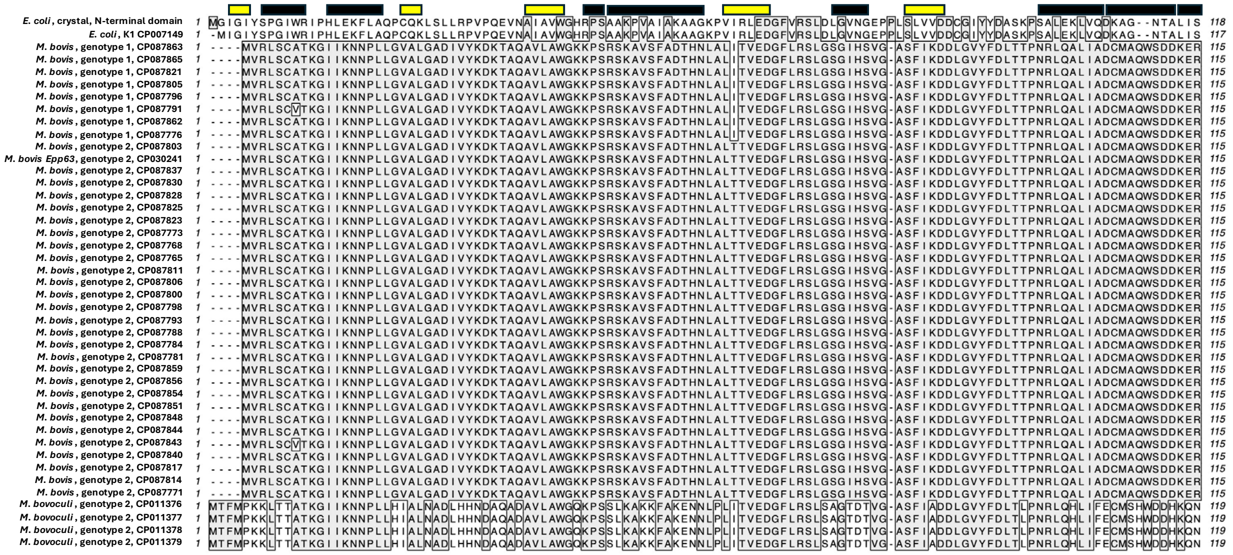

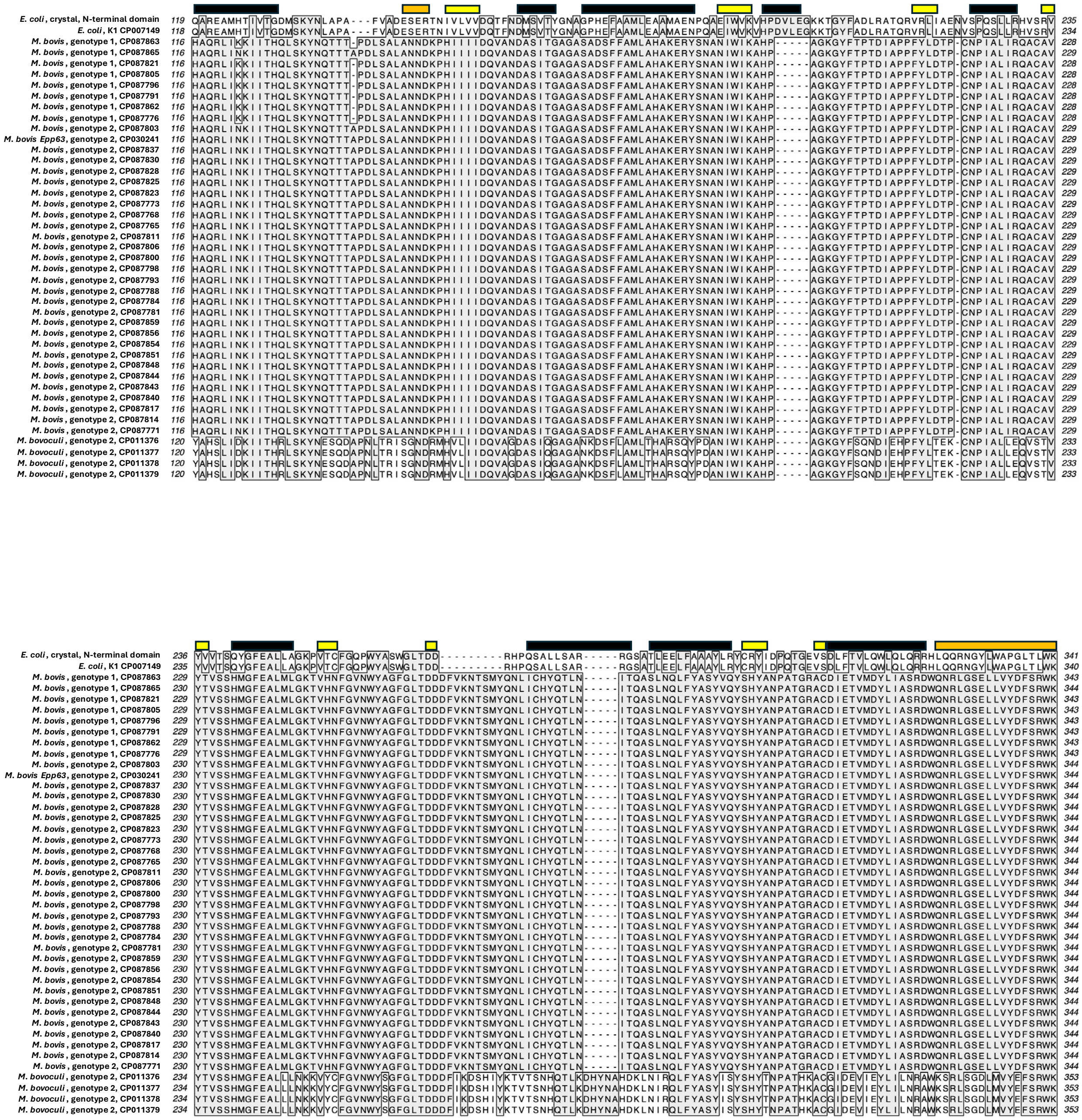

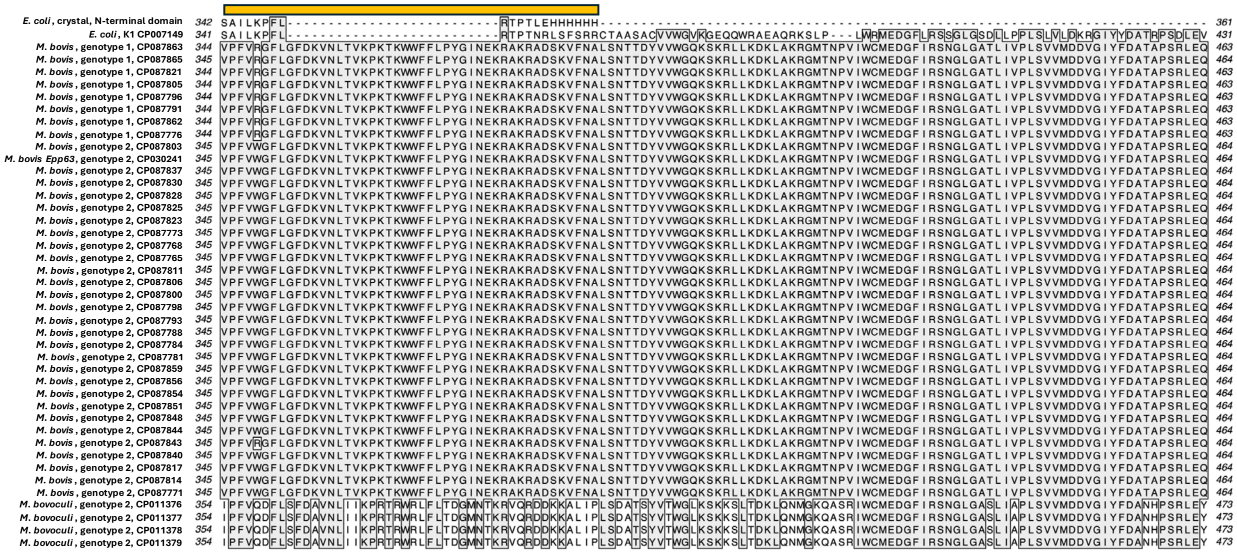

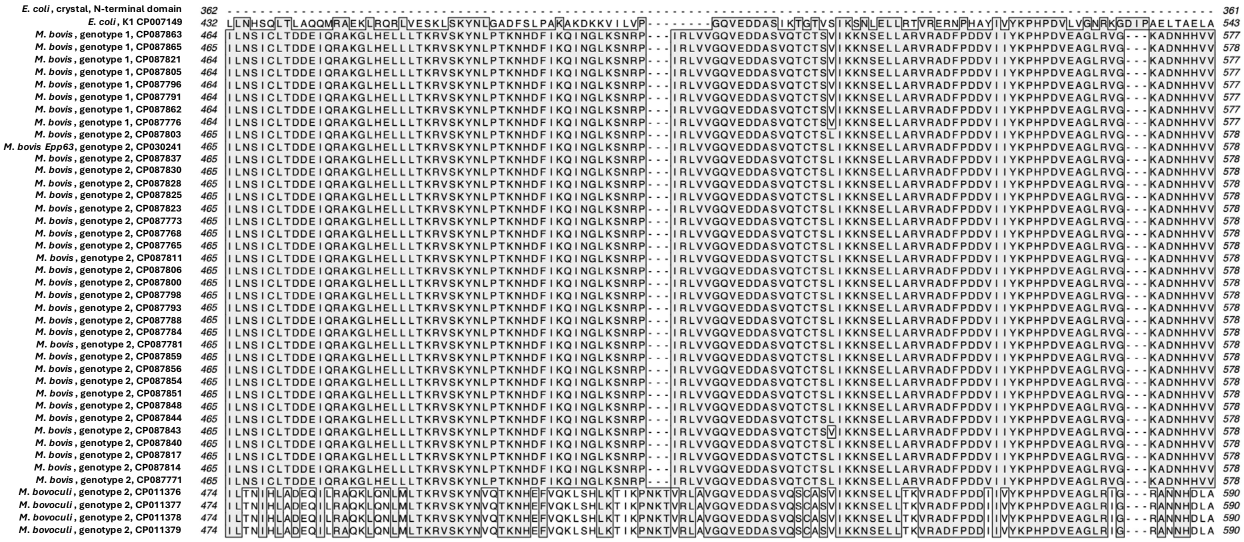

Sequence	Start	Alignment	End
E. coli, crystal, N-terminal domain	362	--	361
E. coli, K1 CP007149	544	DYQALDADIIQCIQRADEVHTMTSLSGFEALLHGKHVHCYGLPFYAGWGLT-------------VDEHRCPRRERKLTLADLIYQALIVYPTYIHPTRLQPITVEEAAEYLIQTPRKPMF	650
M. bovis, genotype 1, CP087863	578	DMVAHDVAMPDCLDVCDVVHTISSLTGFEALLRDKKVVCYGLPFYAGFGLTDDVVESDNTSKINALNRRTRPNLPALTLDGLIYGTLISYPMYHLPHGIGLATPEQVIEYLYNQAHT-AK	696
M. bovis, genotype 1, CP087865	579	DMVAHDVAMPDCLDVCDVVHTISSLTGFEALLRDKKVVCYGLPFYAGFGLTDDVVESDNTSKINALNRRTRPNLPALTLDGLIYGTLISYPMYHLPHGIGLATPEQVIEYLYNQAHT-AK	697
M. bovis, genotype 1, CP087821	578	DMVAHDVAMPDCLDVCDVVHTISSLTGFEALLRDKKVVCYGLPFYAGFGLTDDVVESDNTSKINALNRRTRPNLPALTLDGLIYGTLISYPMYHLPHGIGLATPEQVIEYLYNQAHT-AK	696
M. bovis, genotype 1, CP087805	578	DMVAHDVAMPDCLDVCDVVHTISSLTGFEALLRDKKVVCYGLPFYAGFGLTDDVVESDNTSKINALNRRTRPNLPALTLDGLIYGTLISYPMYHLPHGIGLATPEQVIEYLYNQAHT-AK	696
M. bovis, genotype 1, CP087796	578	DMVAHDVAMPDCLDVCDVVHTISSLTGFEALLRDKKVVCYGLPFYAGFGLTDDVVESDNTSKINALNRRTRPNLPALTLDGLIYGTLISYPMYHLPHGIGLATPEQVIEYLYNQAHT-AK	696
M. bovis, genotype 1, CP087791	578	DMVAHDVAMPDCLDVCDVVHTISSLTGFEALLRDKKVVCYGLPFYAGFGLTDDVVESDNTSKINALNRRTRPNLPALTLDGLIYGTLISYPMYHLPHGIGLATPEQVIEYLYNQAHT-AK	696
M. bovis, genotype 1, CP087862	578	DMVAHDVAMPDCLDVCDVVHTISSLTGFEALLRDKKVVCYGLPFYAGFGLTDDVVESDNTSKINALNRRTRPNLPALTLDGLIYGTLISYPMYHLPHGIGLATPEQVIEYLYNQAHT-AK	696
M. bovis, genotype 1, CP087776	578	DMVAHDVAMPDCLDVCDVVHTISSLTGFEALLRDKKVVCYGLPFYAGFGLTDDVVESDNTSKINALNRRTRPNLPALTLDGLIYGTLISYPMYHLPHGIGLATPEQVIEYLYNQAHT-AK	696
M. bovis, genotype 2, CP087803	579	DMVAHDVAMPDCLDVCDVVHTISSLTGFEALLRDKKVVCYGLPFYAGFGLTDDVVESDNTSKINALNRRTRPNLPALTLDGLIYGTLISYPMYHLPHGIGLATPEQVIEYLYNQAHT-AK	697
M. bovis Epp63, genotype 2, CP030241	579	DMVAHDVAMPDCLDVCDVVHTISSLTGFEALLRDKKVVCYGLPFYAGFGLTDDVVESDNTSKINALNRRTRPNLPALTLDGLIYGTLISYPMYHLPHGIGLATPEQVIEYLYNQAHT-AK	697
M. bovis, genotype 2, CP087837	579	DMVAHDVAMPDCLDVCDVVHTISSLTGFEALLRDKKVVCYGLPFYAGFGLTDDVVESDNTSKINALNRRTRPNLPALTLDGLIYGTLISYPMYHLPHGIGLATPEQVIEYLYNQAHT-AK	697
M. bovis, genotype 2, CP087830	579	DMVAHDVAMPDCLDVCDVVHTISSLTGFEALLRDKKVVCYGLPFYAGFGLTDDVVESDNTSKINALNRRTRPNLPALTLDGLIYGTLISYPMYHLPHGIGLATPEQVIEYLYNQAHT-AK	697
M. bovis, genotype 2, CP087828	579	DMVAHDVAMPDCLDVCDVVHTISSLTGFEALLRDKKVVCYGLPFYAGFGLTDDVVESDNTSKINALNRRTRPNLPALTLDGLIYGTLISYPMYHLPHGIGLATPEQVIEYLYNQAHT-AK	697
M. bovis, genotype 2, CP087825	579	DMVAHDVAMPDCLDVCDVVHTISSLTGFEALLRDKKVVCYGLPFYAGFGLTDDVVESDNTSKINALNRRTRPNLPALTLDGLIYGTLISYPMYHLPHGIGLATPEQVIEYLYNQAHT-AK	697
M. bovis, genotype 2, CP087823	579	DMVAHDVAMPDCLDVCDVVHTISSLTGFEALLRDKKVVCYGLPFYAGFGLTDDVVESDNTSKINALNRRTRPNLPALTLDGLIYGTLISYPMYHLPHGIGLATPEQVIEYLYNQAHT-AK	697
M. bovis, genotype 2, CP087773	579	DMVAHDVAMPDCLDVCDVVHTISSLTGFEALLRDKKVVCYGLPFYAGFGLTDDVVESDNTSKINALNRRTRPNLPALTLDGLIYGTLISYPMYHLPHGIGLATPEQVIEYLYNQAHT-AK	697
M. bovis, genotype 2, CP087768	579	DMVAHDVAMPDCLDVCDVVHTISSLTGFEALLRDKKVVCYGLPFYAGFGLTDDVVESDNTSKINALNRRTRPNLPALTLDGLIYGTLISYPMYHLPHGIGLATPEQVIEYLYNQAHT-AK	697
M. bovis, genotype 2, CP087765	579	DMVAHDVAMPDCLDVCDVVHTISSLTGFEALLRDKKVVCYGLPFYAGFGLTDDVVESDNTSKINALNRRTRPNLPALTLDGLIYGTLISYPMYHLPHGIGLATPEQVIEYLYNQAHT-AK	697
M. bovis, genotype 2, CP087811	579	DMVAHDVAMPDCLDVCDVVHTISSLTGFEALLRDKKVVCYGLPFYAGFGLTDDVVESDNTSKINALNRRTRPNLPALTLDGLIYGTLISYPMYHLPHGIGLATPEQVIEYLYNQAHT-AK	697
M. bovis, genotype 2, CP087806	579	DMVAHDVAMPDCLDVCDVVHTISSLTGFEALLRDKKVVCYGLPFYAGFGLTDDVVESDNTSKINALNRRTRPNLPALTLDGLIYGTLISYPMYHLPHGIGLATPEQVIEYLYNQAHT-AK	697
M. bovis, genotype 2, CP087800	579	DMVAHDVAMPDCLDVCDVVHTISSLTGFEALLRDKKVVCYGLPFYAGFGLTDDVVESDNTSKINALNRRTRPNLPALTLDGLIYGTLISYPMYHLPHGIGLATPEQVIEYLYNQAHT-AK	697
M. bovis, genotype 2, CP087798	579	DMVAHDVAMPDCLDVCDVVHTISSLTGFEALLRDKKVVCYGLPFYAGFGLTDDVVESDNTSKINALNRRTRPNLPALTLDGLIYGTLISYPMYHLPHGIGLATPEQVIEYLYNQAHT-AK	697
M. bovis, genotype 2, CP087793	579	DMVAHDVAMPDCLDVCDVVHTISSLTGFEALLRDKKVVCYGLPFYAGFGLTDDVVESDNTSKINALNRRTRPNLPALTLDGLIYGTLISYPMYHLPHGIGLATPEQVIEYLYNQAHT-AK	697
M. bovis, genotype 2, CP087788	579	DMVAHDVAMPDCLDVCDVVHTISSLTGFEALLRDKKVVCYGLPFYAGFGLTDDVVESDNTSKINALNRRTRPNLPALTLDGLIYGTLISYPMYHLPHGIGLATPEQVIEYLYNQAHT-AK	697
M. bovis, genotype 2, CP087784	579	DMVAHDVAMPDCLDVCDVVHTISSLTGFEALLRDKKVVCYGLPFYAGFGLTDDVVESDNTSKINALNRRTRPNLPALTLDGLIYGTLISYPMYHLPHGIGLATPEQVIEYLYNQAHT-AK	697
M. bovis, genotype 2, CP087781	579	DMVAHDVAMPDCLDVCDVVHTISSLTGFEALLRDKKVVCYGLPFYAGFGLTDDVVESDNTSKINALNRRTRPNLPALTLDGLIYGTLISYPMYHLPHGIGLATPEQVIEYLYNQAHT-AK	697
M. bovis, genotype 2, CP087859	579	DMVAHDVAMPDCLDVCDVVHTISSLTGFEALLRDKKVVCYGLPFYAGFGLTDDVVESDNTSKINALNRRTRPNLPALTLDGLIYGTLISYPMYHLPHGIGLATPEQVIEYLYNQAHT-AK	697
M. bovis, genotype 2, CP087856	579	DMVAHDVAMPDCLDVCDVVHTISSLTGFEALLRDKKVVCYGLPFYAGFGLTDDVVESDNTSKINALNRRTRPNLPALTLDGLIYGTLISYPMYHLPHGIGLATPEQVIEYLYNQAHT-AK	697
M. bovis, genotype 2, CP087854	579	DMVAHDVAMPDCLDVCDVVHTISSLTGFEALLRDKKVVCYGLPFYAGFGLTDDVVESDNTSKINALNRRTRPNLPALTLDGLIYGTLISYPMYHLPHGIGLATPEQVIEYLYNQAHT-AK	697
M. bovis, genotype 2, CP087851	579	DMVAHDVAMPDCLDVCDVVHTISSLTGFEALLRDKKVVCYGLPFYAGFGLTDDVVESDNTSKINALNRRTRPNLPALTLDGLIYGTLISYPMYHLPHGIGLATPEQVIEYLYNQAHT-AK	697
M. bovis, genotype 2, CP087848	579	DMVAHDVAMPDCLDVCDVVHTISSLTGFEALLRDKKVVCYGLPFYAGFGLTDDVVESDNTSKINALNRRTRPNLPALTLDGLIYGTLISYPMYHLPHGIGLATPEQVIEYLYNQAHT-AK	697
M. bovis, genotype 2, CP087844	579	DMVAHDVAMPDCLDVCDVVHTISSLTGFEALLRDKKVVCYGLPFYAGFGLTDDVVESDNTSKINALNRRTRPNLPALTLDGLIYGTLISYPMYHLPHGIGLATPEQVIEYLYNQAHT-AK	697
M. bovis, genotype 2, CP087843	579	DMVAHDVAMPDCLDVCDVVHTISSLTGFEALLRDKKVVCYGLPFYAGFGLTDDVVESDNTSKINALNRRTRPNLPALTLDGLIYGTLISYPMYHLPHGIGLATPEQVIEYLYNQAHT-AK	697
M. bovis, genotype 2, CP087840	579	DMVAHDVAMPDCLDVCDVVHTISSLTGFEALLRDKKVVCYGLPFYAGFGLTDDVVESDNTSKINALNRRTRPNLPALTLDGLIYGTLISYPMYHLPHGIGLATPEQVIEYLYNQAHT-AK	697
M. bovis, genotype 2, CP087817	579	DMVAHDVAMPDCLDVCDVVHTISSLTGFEALLRDKKVVCYGLPFYAGFGLTDDVVESDNTSKINALNRRTRPNLPALTLDGLIYGTLISYPMYHLPHGIGLATPEQVIEYLYNQAHT-AK	697
M. bovis, genotype 2, CP087814	579	DMVAHDVAMPDCLDVCDVVHTISSLTGFEALLRDKKVVCYGLPFYAGFGLTDDVVESDNTSKINALNRRTRPNLPALTLDGLIYGTLISYPMYHLPHGIGLATPEQVIEYLYNQAHT-AK	697
M. bovis, genotype 2, CP087771	579	DMVAHDVAMPDCLDVCDVVHTISSLTGFEALLRDKKVVCYGLPFYAGFGLTDDVVESDNTSKINALNRRTRPNLPALTLDGLIYGTLISYPMYHLPHGIGLATPEQVIEYLYNQAHT-AK	697
M. bovoculi, genotype 2, CP011376	591	DLVASDVAMPDCLDGCDVVHAISSLTGFEALLRGLEVVCYGVPFYAGFGLTTDVVEPDNTAKINALNRRHR--VDGLTLPELIYGVIVEYPLYHLPHGHGLAQPEDVIEYMYNQSSFGVS	708
M. bovoculi, genotype 2, CP011377	591	DLVASDVAMPDCLDGCDVVHAISSLTGFEALLRGLEVVCYGVPFYAGFGLTTDVVEPDNTAKINALNRRHR--VDGLTLPELIYGVIVEYPLYHLPHGHGLAQPEDVIEYMYNQSSFGVS	708
M. bovoculi, genotype 2, CP011378	591	DLVASDVAMPDCLDGCDVVHAISSLTGFEALLRGLEVVCYGVPFYAGFGLTTDVVEPDNTAKINALNRRHR--VDGLTLPELIYGVIVEYPLYHLPHGHGLAQPEDVIEYMYNQSSFGVS	708
M. bovoculi, genotype 2, CP011379	591	DLVASDVAMPDCLDGCDVVHAISSLTGFEALLRGLEVVCYGVPFYAGFGLTTDVVEPDNTAKINALNRRHR--VDGLTLPELIYGVIVEYPLYHLPHGHGLAQPEDVIEYMYNQSSFGVS	708

Sequence	Start	Alignment	End
E. coli, crystal, N-terminal domain	362	-------------------------	361
E. coli, K1 CP007149	651	ITRKKAGRVIRYYRKLIMFCKVRFG	675
M. bovis, genotype 1, CP087863	697	PSLAKRLTQNVKTTAMQLRKIIIR-	720
M. bovis, genotype 1, CP087865	698	PSLAKRLTQNVKTTAMQLRKIIIR-	721
M. bovis, genotype 1, CP087821	697	PSLAKRLTQNVKTTAMQLRKIIIR-	720
M. bovis, genotype 1, CP087805	697	PSLAKRLTQNVKTTAMQLRKIIIR-	720
M. bovis, genotype 1, CP087796	697	PSLAKRLTQNVKTTAMQLRKIIIR-	720
M. bovis, genotype 1, CP087791	697	PSLAKRLTQNVKTTAMQLRKIIIR-	720
M. bovis, genotype 1, CP087862	697	PSLAKRLTQNVKTTAMQLRKIIIR-	720
M. bovis, genotype 1, CP087776	697	PSLAKRLTQNVKTTAMQLRKIIIR-	720
M. bovis, genotype 2, CP087803	698	PSLAKRLTQNVKTTAMQLRKIIIR-	721
M. bovis Epp63, genotype 2, CP030241	698	PSLAKRLTQNVKTTAMQLRKIIIR-	721
M. bovis, genotype 2, CP087837	698	PSLAKRLTQNVKTTAMQLRKIIIR-	721
M. bovis, genotype 2, CP087830	698	PSLAKRLTQNVKTTAMQLRKIIIR-	721
M. bovis, genotype 2, CP087828	698	PSLAKRLTQNVKTTAMQLRKIIIR-	721
M. bovis, genotype 2, CP087825	698	PSLAKRLTQNVKTTAMQLRKIIIR-	721
M. bovis, genotype 2, CP087823	698	PSLAKRLTQNVKTTAMQLRKIIIR-	721
M. bovis, genotype 2, CP087773	698	PSLAKRLTQNVKTTAMQLRKIIIR-	721
M. bovis, genotype 2, CP087768	698	PSLAKRLTQNVKTTAMQLRKIIIR-	721
M. bovis, genotype 2, CP087765	698	PSLAKRLTQNVKTTAMQLRKIIIR-	721
M. bovis, genotype 2, CP087811	698	PSLAKRLTQNVKTTAMQLRKIIIR-	721
M. bovis, genotype 2, CP087806	698	PSLAKRLTQNVKTTAMQLRKIIIR-	721
M. bovis, genotype 2, CP087800	698	PSLAKRLTQNVKTTAMQLRKIIIR-	721
M. bovis, genotype 2, CP087798	698	PSLAKRLTQNVKTTAMQLRKIIIR-	721
M. bovis, genotype 2, CP087793	698	PSLAKRLTQNVKTTAMQLRKIIIR-	721
M. bovis, genotype 2, CP087788	698	PSLAKRLTQNVKTTAMQLRKIIIR-	721
M. bovis, genotype 2, CP087784	698	PSLAKRLTQNVKTTAMQLRKIIIR-	721
M. bovis, genotype 2, CP087781	698	PSLAKRLTQNVKTTAMQLRKIIIR-	721
M. bovis, genotype 2, CP087859	698	PSLAKRLTQNVKTTAMQLRKIIIR-	721
M. bovis, genotype 2, CP087856	698	PSLAKRLTQNVKTTAMQLRKIIIR-	721
M. bovis, genotype 2, CP087854	698	PSLAKRLTQNVKTTAMQLRKIIIR-	721
M. bovis, genotype 2, CP087851	698	PSLAKRLTQNVKTTAMQLRKIIIR-	721
M. bovis, genotype 2, CP087848	698	PSLAKRLTQNVKTTAMQLRKIIIR-	721
M. bovis, genotype 2, CP087844	698	PSLAKRLTQNVKTTAMQLRKIIIR-	721
M. bovis, genotype 2, CP087843	698	PSLAKRLTQNVKTTAMQLRKIIIR-	721
M. bovis, genotype 2, CP087840	698	PSLAKRLTQNVKTTAMQLRKIIIR-	721
M. bovis, genotype 2, CP087817	698	PSLAKRLTQNVKTTAMQLRKIIIR-	721
M. bovis, genotype 2, CP087814	698	PSLAKRLTQNVKTTAMQLRKIIIR-	721
M. bovis, genotype 2, CP087771	698	PSLAKRLTQNVKTTAMQLRKIIIR-	721
M. bovoculi, genotype 2, CP011376	709	IPWQSKMWQSIKTNAMRLHKFTKQ-	732
M. bovoculi, genotype 2, CP011377	709	IPWQSKMWQSIKTNAMRLHKFTKQ-	732
M. bovoculi, genotype 2, CP011378	709	IPWQSKMWQSIKTNAMRLHKFTKQ-	732
M. bovoculi, genotype 2, CP011379	709	IPWQSKMWQSIKTNAMRLHKFTKQ-	732

Figure S3. Multiple alignment of *M. bovis* and *M. bovoculi* sequences with protein sequence of *E. coli* K4 chondroitin synthase.

Labels in the alignment show GenBank accession numbers for the sequences. The alignment contains chondroitin synthase sequence from the crystalized *E. coli* kpsC (K4CP). Sheet and helix motifs of the crystal structure are represented with yellow and black blocks, respectively. Unmodeled residues in the crystal structure are represented with orange blocks.

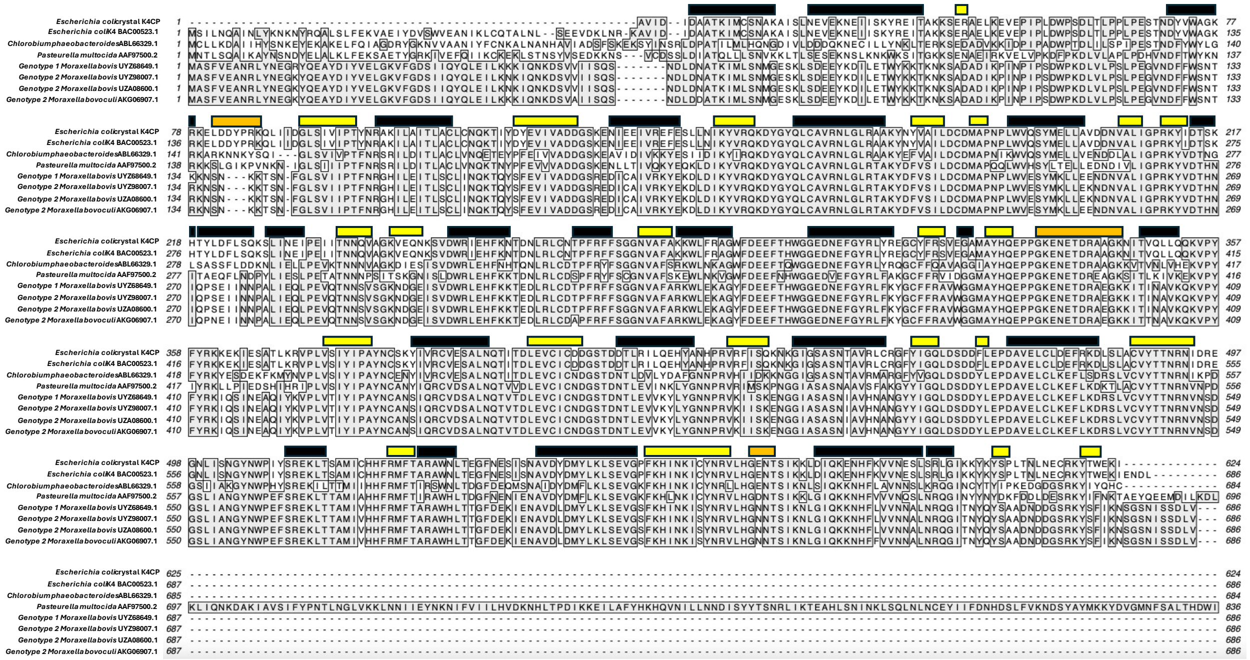

Supplement: Supplementary file 1 [file id5c00628_si_001.pdf]
